# Statin-related Muscle Toxicity: An Evidence-based Review

**DOI:** 10.17925/EE.2022.18.2.89

**Published:** 2022-11-21

**Authors:** Mohammad S Jeeyavudeen, Joseph M Pappachan, Ganesan Arunagirinathan

**Affiliations:** 1. Western General Hospital, Edinburgh Centre for Endocrinology Diabetes, Edinburgh, UK; 2. Lancashire Teaching Hospitals NHS Trust, Lancashire, UK; 3. Manchester Metropolitan University, Manchester, UK

**Keywords:** Cardiovascular risk, creatine kinase, statin-associated muscle symptoms, statin-related muscle toxicity, statins

## Abstract

The efficacy of statins in the primary and secondary prevention of cardiovascular disease has been proven beyond doubt. The number needed to treat to prevent one cardiovascular event is 1 in 30 over 10 years, and the number needed to treat for secondary prevention is much lower. However, a recent study demonstrated that only 68% of eligible patients are on statin therapy. Moreover, there seems to be a reluctance to escalate statin doses due to the fear of adverse effects. The adverse effects that worries patients and their physicians most frequently are those related to muscular symptoms. N-of-1 trial evidence suggests that muscular symptoms attributed to statins are often caused by the nocebo effect. This article aims to provide a structured, evidence-based approach to suspected statin-related muscle toxicity.

In the early 1970s, the discovery of statin by Dr Akira Endo changed the fate of cardiovascular disease prevention and the treatment of atherosclerosis. It was during this period that the ratelimiting step in cholesterol biosynthesis was revealed, and the enzyme hydroxy-3-methylglutaryl-coenzyme A (HMG-CoA) reductase became the target for many pharmaceutical products. Brought up in a farming family in Japan and educated by his grandfather, Dr Endo’s main research interest was fungi. His keen observation skills and attention to detail led him to note that the broth of bacterium closer to the fungal colony was inhibited without causing much damage to its own fungal growth.^1^ This remarkable observation led Dr Endo to the reasonable conclusion that a byproduct of fungal metabolism inhibited a crucial step in cholesterol biosynthesis, causing the destruction of the bacterial cell membrane without damaging its own fungal cell wall, which is composed of ergosterol rather than cholesterol. More than 5,000 different species of fungal broth were tested, and finally, in late August 1973, a compound called compactin (ML236B or mevastatin) was isolated from the fungal broth of *Penicillium citrinum*; it was the first and one of the most potent HMG-CoA reductase inhibitors to be identified.^2^ This led to significant interest from various pharmaceutical companies and to the investigation of a plethora of fungal species in clinical trials of various cholesterol pathways and their effects on serum cholesterol level.^3^

The compound mevinolin (later named lovastatin by Merck Pharmaceuticals) was discovered in 1978 from the broth of *Aspergillus terreus,* and its effect was studied for its cholesterol-lowering properties by the inhibition of the HMG-CoA reductase enzyme.^4^ In the early 1980s, numerous clinical and animal trials reported the property of lovastatin to significantly low-density lipoprotein cholesterol (LDL-C) without major adverse clinical effects.^5^ These findings resulted in lovastatin gaining US Food and Drug Administration approval in August 1987. The success of lovastatin prompted the development of several more statin variants. In 1988, the next statin to become available was simvastatin, which is a semi-synthetic derivative of lovastatin, differing from the parent compound by the addition of a methyl moiety side chain.^6^ As more and more data on the LDL-C-lowering and cardiovascular protective properties emerged, several different statins with good side effect profiles were introduced into the market. The four commonly used statins now in clinical practice are atorvastatin, simvastatin, rosuvastatin and pravastatin.^7^

Statins have been shown conclusively to reduce the cardiovascular risk; the number needed to treat to prevent one cardiovascular event is 1 in 30 over 10 years, and the number needed to treat for secondary prevention is much lower.^8^ However, there seems to be a reluctance among patients to start or continue statin therapy, with a recent study demonstrating that only 68% of eligible patients are on statin therapy, which is thought to be partly due to bad media publicity.^9^ One of the reasons for this behaviour is the muscle side effect profile of statins.^10^ However, recent evidence from N-of-1 trial data demonstrates that the risk of statin-associated muscular symptoms (SAMS) is potentially very small when compared with the benefits of reduced cardiovascular risk.^11^ This evidence-based review aims to provide a more accurate picture of the relationship between statin therapy and statin-related myotoxicity (SRM) with a structured approach to dealing with muscular symptoms when they occur.

**Table 1:** Statin-related muscle toxicity phenotypic categories^23–40^

SRM classification	Phenotype	Incidence	Definition	Reference
SRM 0	CK elevation <4x ULN	1.5–26%	No muscle symptoms	Refs 24, 28, 31, 32, 37
SRM 1	Myalgia, tolerable	190/100,000 Patient-years; 0.3–33%	Muscle symptoms without CK elevation	Refs 24, 27, 29, 35, 38
SRM 2	Myalgia, intolerable	0.2–2/1,000	Muscle symptoms, CK <4x ULN, complete resolution on dechallenge	Ref 28
SRM 3	Myopathy	5/100,000 Patient-years	CK elevation >4x ULN <10x ULN ± muscle symptoms, complete resolution on dechallenge	Ref 24
SRM 4	Severe myopathy	0.11%	CK elevation >10x ULN <50x ULN, muscle symptoms, complete resolution on dechallenge	Refs 28, 39
SRM 5	Phabdomyolosis	0.1–8.4/100,000 Patient-years	CK elevation >10x ULN with evidence of renal impairment + muscle symptoms or CK >50x ULN	Refs 25, 26, 30, 33, 34
SRM 6	Autoimmune-mediated necrotizing myositis	∼2/million per year	HMGCR antibodies, HMGCR expressions in muscle biopsy, incomplete resolution on dechallenge	Refs 36, 40

*Reproduced wiht permission from Alfirevic et al. (2014).^23^*

*CK = creatine kinase; HMGCR = hydroxy-3-methylglutaryl-coenzyme A reductase; SRM = statin-related myotoxicity; ULN = upper limit of normal.*

## Classifications of statins

Although, as a drug class, statins reduce the LDL-C, individual statins vary in their potency and bio-distribution. The polar moiety on the statin hydrophobic backbone defines the solubility state of the statin in the phospholipid bilayer.^12^ The predominantly lipophilic statins are fluvastatin, atorvastatin, simvastatin, lovastatin and pitavastatin, which readily pass through the cell membranes, where they interact with surrounding acyl chains. In contrast, the more hydrophilic agents are pravastatin and, to a lesser degree, rosuvastatin, which tend to be associated with the polar surface of the cell membrane and require transport proteins to gain cellular entry in order to inhibit the HMG-CoA reductase enzyme.^7^ As lipophilic statins have greater tissue permeability and can passively diffuse into the myotubules, there is a tendency towards more SAMS with these agents. *In vitro* studies have demonstrated a greater generation of myotubular degradation products with similar concentrations of simvastatin and atorvastatin but only at higher concentrations of rosuvastatin.^13^ This property can be used in some patients when they develop SAMS by switching from a lipophilic statin to a hydrophilic agent, although this strategy may not be universally successful.^14^

## Statins in the prevention of cardiovascular disease

### Primary prevention

A recent large meta-analysis by Yebyo et al. in 2019^15^ revealed that the risk of non-fatal myocardial infarction was significantly reduced with statins as a class effect (relative risk: 0.62; 95% confidence interval [CI]: 0.53–0.72) compared with placebo. In a drug-level network analysis, statins that showed a significant reduction in non-fatal myocardial infarction events were atorvastatin, rosuvastatin and pravastatin, but not lovastatin. The most effective treatment outcomes were observed with atorvastatin, and, hence, it is one of the most prescribed statins.

### Secondary prevention

High-intensity statin therapy is recommended for the secondary prevention of atherosclerotic cardiovascular disease (ASCVD) and also for individuals who do not achieve a 40% reduction of baseline non-high-density lipoprotein-cholesterol level when used in low or moderate doses for primary prevention.^16^ The dose of statin defined as high intensity is based on its ability to reduce the LDL-C to 50% of the baseline level. Of the commonly used statins, the daily dose of atorvastatin 40–80 mg or rosuvastatin 20–40 mg is characterized as a high-intensity dosage.^17^ Moreover, in patients at very high risk, the absolute value of LDL-C levels ≥70 mg/dL on maximally tolerated statin therapy or in patients who do not tolerate high-intensity statin therapy, the addition of ezetimibe, bempedoic acid or proprotein convertase subtilisin/kexin 9 (PCSK9) inhibitor is beneficial, as these agents have further LDL-C-lowering effects that are independent of statin.^18^ The dosage needs to be reviewed in frail older patients, in whom the benefit may outweigh the risk for muscle toxicity.^19^

## Statin-related adverse effects

The most common adverse effects associated with statins involve muscle-related toxicity, which results in myalgia, myopathy and rhabdomyolysis.^20^ Less common adverse effects include hepatotoxicity, the incidence of new-onset type 2 diabetes mellitus or worsening of preexisting diabetes mellitus, neurological side effects, and gastrointestinal symptoms. A detailed review of all statin-related adverse effects is beyond the scope of this review, which focuses on SRM.

## Defining and quantifying statin-related muscle toxicity

Although there are various definitions of statin intolerance available in the literature, the two most commonly used definitions are from the National Lipid Association of the United States and the European Atherosclerosis Society. The National Lipid Association defines statin intolerance as the “inability to tolerate at least two statins: One statin at the lowest starting daily dose and another statin at any daily dose, due to either objectionable symptoms (real or perceived) or abnormal laboratory determinations, which are temporally related to statin treatment and reversible upon statin discontinuation”.^21^ The European Atherosclerosis Society states that “the probability of SAMS being due to a statin take account of the nature of the muscle symptoms, the elevation in CK [creatine kinase] levels and their temporal association with statin initiation, discontinuation, and re-challenge”.^22^ For patients taking statins, muscular symptoms attributed to statin therapy are referred to as SRM, whereas cases of muscle symptoms unrelated to a statin are described as non-SRM. SRM can be classified based on the severity of the muscle symptoms and the height of cretine kinase (CK) elevation into seven distinct phenotypic categories (*Table 1*).^23–40^

## Statin and the nocebo effect

The incidence of SRM varies across studies due to differing definitions used in various nations, as well as the difficulty in differentiating between true SRM and a nocebo effect. The term ‘nocebo effect’ was coined in 1961 to explain the adverse symptoms reported with placebo medications used in clinical trials.^23,41^ In the case of statins, some argue that, as statins, compared with the inert placebo medication, contain active drug component, the term ‘drucebo effect’ is more appropriate.^42^ The mechanism underlying the nocebo effect is complex and is not as well understood as the placebo effect.^41^ When categorized into SRM, the incidence of SRM, as expected, is most common for SRM 0 (1.5–26%) compared with SRM 6, which occurs in only two out of every million statin users every year.^23,43^ Intermediate categories vary in prevalence and are influenced by the presence of other risk factors and individual predispositions. In the SAMSON n-of-1 trial (Self-assessment method for statin side-effects or nocebo; ClinicalTrials.gov identifier: NCT02668016), the participants who developed muscle side effects were re-challenged with alternating statin and placebo each month.^11,44^ Symptoms were monitored with a mobile smartphone application. There was no significant difference in symptom severity between the months on statin versus those on placebo. Similar results were also observed in the StatinWISE study (STATIN: Web-based investigation of side effects; ClinicalTrials.gov identifier: NCT02781064), although StatinWISE did not include a placebo arm.^45^

**Figure 1:**
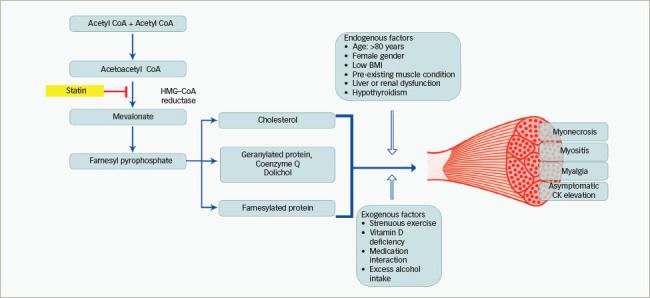
Pathogenesis and risk for statin-induced muscle damage

*BMI = body mass index; CK = creatine kinase; CoA = coenzyme A; HMG-CoA = hydroxy-3-methylglutaryl-coenzyme A.*

## Pathogenesis of statin-related muscle toxicity

Numerous risk factors and pathological mechanisms have been proposed for the development of SRM, but a detailed mechanism has yet to be determined. Two main interdependent mechanisms have been studied:

- increased systemic statin exposure due to pharmacodynamic, pharmacokinetic and pharmacogenomic factors
- statin gaining intracellular skeletal myocyte access and dysregulation of muscle function.

### Risk factors implicated in the pathogenesis of statin-related muscle toxicity

Risk factors for SRM have been broadly classified into endogenous and exogenous factors. Endogenous factors include advanced age (>80 years old), genetic risk factors, ethnicity, female sex, pre-existing neuromuscular disorder, hypothyroidism, liver or renal failure and low body mass index (*Figure 1*).^46^ Exogenous factors include excess alcohol intake, vitamin D deficiency, strenuous exercise and drug interactions. Pitavastatin, simvastatin and atorvastatin are predominantly lipophilic statins with first-pass metabolism in the liver, catalysed through cytochrome P450 3A4 reaction. Female individuals have high cytochrome P450 3A4 activity, which theoretically should make them less prone to SRM, as statin will be cleared from the system very easily. Similarly, drugs and food substances that can interact with the cytochrome P450 system can alter the individual risk for SRM.^47^

Asian people are highly susceptible to SRM. In a large retrospective survey of Japanese patients with ASCVD or diabetes mellitus started with either statin or ezetimibe therapy, a higher discontinuation rate of 32.9% within 12 months after starting therapy was noted; moreover, true statin intolerance was seen in only about 10% of the patients.^48^ Data from the SEARCH randomized controlled trial (Study of the effectiveness of additional reductions in cholesterol and homocysteine; ISRCTN identifier: ISRCTN74348595) revealed that the presence of chronic kidney damage is a major risk factor for SRM owing to reduced clearance and is especially seen with high-dose simvastatin.^32^ Statins are reversible blockers for the HMG-CoA reductase enzyme, thereby reducing cholesterol, 7-dehydrocholesterol and vitamin D production. In the study by Pennisi et al.,^49^ the authors found a significant association between low vitamin D status and SRM, with a sensitivity of 77% (95% CI: 71.6–81.7%) and specificity of 63.4% (95% CI: 60.2–66.5%) for diagnosing SRM when the vitamin D levels were <30 nmol/L.

Hypothyroidism alone can produce muscle weakness and myopathy with CK elevation.^50^ The exact pathogenesis of thyroid myopathy is unclear, and mechanisms postulated are the altered energy handling in the tissue due to impaired glycogenolysis and mitochondrial dysfunction. As type II muscle fibres predominantly depend on glycogenolysis, impairment of this process will lead to muscle degeneration and atrophy.^52^ A compensatory hypertrophy of the type I muscle fibres is seen in severe long-standing cases, leading to pseudohypertrophy of the muscle group. Statin-mediated reduction of coenzyme Q generation may cause added mitochondrial stress. Severe cases of rhabdomyolysis have been reported with statin exposure in patients with hypothyroidism and complete resolution of the CK after achieving a euthyroid state.^50^

Genetic variations represent a further risk factor for the development of SRM. Polymorphism in the *SLCO1B1* gene on chromosome 12 that codes for a hepatic transport protein OATP1B1 (organic anion transporting polypeptide 1B1) alters the uptake of statins from the circulation. People with the c.521CC genotype have three times the level of the simvastatin metabolite simvastatin acid compared with those with the reference c.521TT genotype.^52^ Genome-wide association studies have explored the associations between different genotypes and SRM, and data from the SEARCH randomized controlled trial further confirmed the association with the *SLCO1B1* c.521C variant.^32^ Presence of the c.521C variant has 4.5 per copy for the C allele and 16.9 per copy for the CC genotype for the development of SRM compared with the reference TT genotype. A higher risk for SRM with the c.521C variant was seen only with simvastatin and not for any other statin. Furthermore, the *SLCO1B1* variant accounts for a lower proportion of cases of SRM when compared with the overall occurrence of SRM, although the association is not consistent.^52^

### Molecular pathway of statin-related muscle toxicity

Statins block the mevalonate pathway, which is vital for lipoprotein and cell membrane regulation and skeletal muscle adaptation. Also inhibited is the synthesis of dolichols, ubiquinone and prenylated proteins. Skeletal muscle cellular protein degradation and architecture rely on the ubiquinone level, which is a key component of the ubiquitin–proteasome pathway.^53^ Furthermore, the synthesis of glycoprotein for myofibrillar growth relies heavily on the dolichols (*Figure 1*). Cholesterol *per se* is needed for the synthesis of the cell membrane, which in turn is inhibited by statin. Hence, statins not only reduce the key component of muscle membrane synthesis but also, in turn, prevent the cellular degradation pathway that clears intracellular debris, resulting in toxin accumulation and free oxygen-mediated cellular damage.^54^ Coenzyme Q10, which maintains the integrity of the mitochondrial function, and isoprenoids, which are important in preventing apoptosis, are byproducts of the cholesterol pathway and are also reduced with statin therapy.^55^ The effect of statin on smooth muscle is unclear, with evidence showing varying results. Lovastatin induces minimal programmed cell death in medial smooth muscle vascular cells, whereas intimal cells showed a pronounced rate of apoptosis.^56^ The transcriptional activation of atrial cell mitochondrial biogenesis has been seen in statin users, and this probably increases the antioxidant reserve and decreases the negative cardiac myocardial remodelling.^57^ In an extremely rare scenario, statins can upregulate the production of the anti-HMG-CoA reductase (anti-HMGCR) antibody, thus causing necrotic damage to the muscle fibres and resulting in a condition described as statin-induced necrotizing autoimmune myositis (SINAM). CK levels are very high (50–100 times) in SINAM, and the large amounts of myoglobin released from the muscle enter the circulation and get filtered in the glomerulus to be deposited in the renal tubules, which can result in acute kidney injury.^58^ As SINAM is an antibody-mediated process, it differs from other SRMs, as symptoms may occur months after stopping statin therapy. Immunosuppressive medications play a key role in the treatment of SINAM.^58^ The exact pathogenesis of SINAM is unclear, with some reports suggesting overexpression of HMG-CoA reductase in affected patients.

## Diagnosis of statin-related muscle toxicity

Thorough history taking to determine the site, distribution and onset of muscle symptoms with temporal correlation to the initiation of statin therapy will provide an important clue to the diagnosis of SRM. Classically, the symptoms are exertional and occur in the bulkier muscle groups when the muscle is put into action. In over 90% of patients, SRM usually happens within 3 months from the start of statin therapy; moreover, if the patients develop muscle symptoms outside of this time window, a search for non-statin SRM causes is warranted.^55^ The onset also varies with the type of statin: with pitavastatin, SRM occurs as early as 14 days, whereas, with pravastatin and fluvastatin, SRM will usually take at least 40–45 days to manifest.^59^ It is also important to understand the interaction of statins with other commonly used medications, such as the interaction between simvastatin and calcium channel blockers, macrolide antibiotics and protease inhibitors, and with food substances such as grapefruit juice consumed in very high quantities.^60^ Hence, a history of non-statin drug initiation and a change in the dietary pattern also need to be considered for accurately diagnosing SRM, as in these circumstances, stopping the culprit agent without stopping statin will resolve the SRM without causing added risk for ASCVD progression.

The Statin-Associated Muscle Symptom Clinical Index (SAM-CI) is a validated, comprehensive clinical score that takes into account the distribution of the muscle involved and the temporal correlation with symptoms on challenge, de-challenge and re-challenge of statins.^61^ A score of <7 indicates that SAMS is less likely. Other clinical indices are available, but these have not been fully validated and can be cumbersome to apply in busy daily practice.^61^ CK level is the cornerstone in the assessment of the severity of SRM. Although muscle symptoms can happen without the elevation of CK, the diagnosis is likely when the CK level elevation regresses after withdrawal of statin therapy and becomes elevated again on re-challenge. Most guidelines recommend routine measurement of CK from 4 weeks to 3 months after the initiation of statin therapy to check for asymptomatic muscle damage.^22^ Muscle biopsy is less useful in classical cases of SRM but may be warranted in patients to rule out a primary muscle disorder that might be unmasked when statin therapy is started.^62^

CK assay results need to be standardized, as the CK level tends to vary between different ethnic, sex and age groups. Black men of African-Caribbean descent tend to have a higher baseline CK level compared with white men of the same age, and this has been attributed to high enzyme activity with passive diffusion across the sarcolemma membrane.^63^ In patients with myalgia and myopathy (SRM 2 and 3, respectively), either the CK level is not raised, or if it is elevated, then it is usually less than four times the upper limit of normal (ULN). CK levels in severe myopathy and rhabdomyolysis (SRM 4 and 5, respectively) are raised above 10 times and 50 times the ULN, respectively (see *Table 1*). The higher the CK level, the greater the risk for acute metabolic complications, and with a greater release of myoglobin in severe muscle necrosis, the chance of acute kidney injury is very high. Hence, CK levels should be urgently checked in patients complaining of muscle symptoms or highly coloured urine (suggestive of myoglobinuria) in order to avoid severe adverse effects, despite their occurrence being very low.^17^

Electrophysiological tests such as electromyography and nerve conduction velocity (NCV) are less useful in the diagnosis of SRM, as the changes due to muscle damage are non-specific. In a study of 25 patients on atorvastatin, the muscle fibre conduction velocity was reduced in statin users, with a more significant reduction in patients with diabetes.^64^ On the other hand, electromyography and nerve conduction velocity have their place when non-SRMs, such as primary myopathies or neuromuscular disorders, are considered the cause of muscle symptoms.^46^ No imaging modality is specific for SRM, hence it is not useful in routine clinical practice. In retrospective data from 21 patients with myopathy due to lipid-lowering therapy (statin and fibrates), the intensity of the muscle damage showed a good correlation with the degree of CK elevation.^65^ In extremely rare cases of focal myositis due to statin, these imaging modalities can be used.^66^

**Figure 2:**
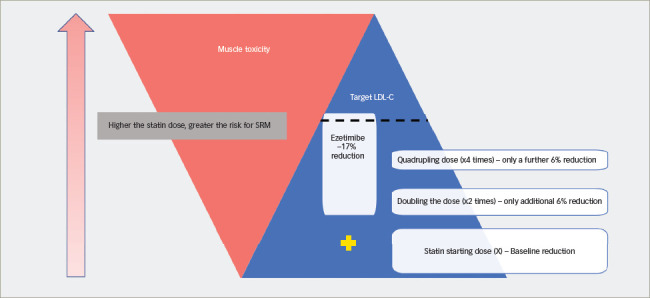
Rule of six. Doubling the statin dose reduces the low-density lipoprotein cholesterol (LDL-C) by only 6% compared with the addition of ezetimibe, which causes 17% LDL-C reduction with no added risk for statin-related muscle toxicity and better chance of achieving the target LDL-C

*LDL-C = low-density lipoprotein cholesterol; SRM = statin-related muscle toxicity.*

More invasive diagnostic tests, such as muscle biopsy, have no added value in typical cases of SRM. Muscle biopsy in SRM shows varying degrees of reduction in the size of the myofibres and degree of inflammatory cellular infiltrate.^67^ Immunohistochemical staining can show positive expression of Bcl-2 in patients with CK elevation on statins, whereas p53 staining was mostly negative, with rare positivity among statin users with CK elevation; however, the significance of these findings needs to be confirmed by a larger study to confirm it.^64^ Hence, a biopsy is only recommended in non-SRM or in atypical cases when there is a failure of resolution of CK even after stopping statin therapy.^46^

## Management strategies for statin-induced myalgia

A structured approach to the management of SRM can prevent the misdiagnosis of the nocebo effect as SRM and reduce the period that the patient is not on statins, especially in patients at very high risk of ASCVD, as a disruption of the therapy, even for only 4 weeks, can cause the instability of the atherosclerotic plaque.^67^ Patients at risk of the nocebo effect include those with depression, anxiety and a tendency toward somatization, those with baseline muscle symptoms or those who had already experienced the nocebo effect with a different medication.^41,43^ These patients should have a well-balanced and structured discussion about the potential benefits of statin therapy compared with its side effects. Patients should also be properly counselled and directed to reliable information on the internet, as misinformation from various sources can confuse patients, potentially resulting in the discontinuation of the medication. Hence, the clinician should take the utmost care with at-risk individuals to prevent the precipitation of SRM or the nocebo effect. If a patient develops SRM, then an individualized management plan should be adopted based on the severity of SRM and cardiovascular risk.

The International Lipid Expert Panel recommends the MEDS approach for the management of SRM.^42^ In the MEDS approach, the time off statins is *minimized*; the patient is informed about the risks and benefits of statin therapy through proper *education*; *diet* or nutraceuticals are added to keep the statin dose to a minimum to prevent SRM; and, finally, monitoring of biomarkers and *symptoms* are monitored to facilitate early identification of SRM.

### Asymptomatic patients with creatine kinase elevation

CK elevation is increasingly encountered as routine CK monitoring is now advocated in most national guidelines.^17,18^ A minor elevation of CK (less than four times the ULN) can often be managed with the continuation of statin therapy and careful monitoring of CK levels. More significant elevation above this limit warrants the discontinuation of the therapy and switching to an alternative lipid-lowering agent in high-risk individuals to maintain the LDL-C levels.^42^ Following discontinuation, any symptoms should be allowed to resolve if present, and when the CK level returns to normal, either the same drug regimen or a lower dose of the same drug(s) can be used, as higher doses are associated with a greater risk for SRM.^54^

### Management of statin-induced myalgia and non-significant creatine kinase elevation

It is safe to continue statin therapy in SRM 1 and 2 with CK less than four times the ULN, as long as the patient can tolerate the on-going therapy, as this reflects the varying degree of nocebo/drucebo effect due to statins.^42^ However, if the symptoms are severe, then statins are discontinued and re-challenged once the symptoms resolve. Different strategies have been tried to prevent the re-occurrence of symptoms, such as keeping the dose as low as possible, using alternate-day statin treatment or using an alternative statin with a reduced muscle side effect profile, such as pravastatin or fluvastatin.^68^ In patients at high or very high risk of ASCVD, during the re-challenge period, other non-myotoxic lipid-lowering agents should be started to prevent ASCVD events.^62^

### Management of statin-induced myositis and significant creatine kinase elevation

In patients with a CK elevation of more than four times the ULN, and definitely in those with a CK elevation of above 10 times the ULN, even without the muscle symptoms, statin therapy should be stopped. This usually happens in patients who are on high-dose statin therapy; however, these are also the patients with a high risk for ASCVD. An alternative muscle-safe lipid-lowering agent such as ezetimibe, bempedoic acid, PCSK9 inhibitor or inclisiran should be started until the resolution of the symptoms and CK level. In the IMPROVE-IT trial (IMPROVE-IT: Examining outcomes in subjects with acute coronary syndrome: VYTORIN [ezetimibe/simvastatin] vs simvastatin [P04103]; ClinicalTrials.gov identifier: NCT00202878), ezetimibe showed a significant reduction of LDL-C in patients who had discontinued statin due to SRM.^69^ Moreover, the combination of simvastatin with ezetimibe (10 mg) led to an additional 17% LDL-C reduction compared with the doubling of statin dose, which reduced the LDL-C level only by 6% but with a greater risk of SRM occurrence (*Figure 2*).^47,52,58^ From the clinical endpoint, there was also a reduction of myocardial infarction in the combination arm, but the overall mortality and mortality due to cardiovascular disease showed no significant reduction.^69^ If a maximally tolerated statin–ezetimibe combination does not achieve the target effect, then PCSK9 inhibitors such as the monoclonal agents evolocumab or alirocumab, or small molecule RNA therapy in the form of inclisiran may be considered. Complex cases may merit a referral to a lipid clinic. Lipoprotein apheresis may be considered if drug therapies are insufficiently effective or inappropriate due to adverse effects but tends to be reserved for individuals with familial hypercholesterolaemia and known ASCVD.^42^ Variable results are reported within the literature for coenzyme Q10 and vitamin D as treatments for SAMS. However, as their effect is not supported by robust randomized trials, they cannot be recommended routinely.^47^

### Management of statin-induced necrotizing myositis

SINAM is an exceptionally uncommon condition, with an occurrence of 1–2 cases per million statin users in a year.^58^ Diagnosis is based on the extreme elevation of CK level and the presence of anti-HMGCR antibody. It is a self-limiting autoimmune process triggered by statins, and hence cessation of therapy in most circumstances usually resolves the inflammation and results in the normalization of the CK level.^42^ In some cases, myonecrosis can be self-sustained despite statin withdrawal, and then the use of immunomodulatory agents is warranted. Due to the rarity of SINAM, there are no randomized controlled trials to suggest one immunosuppressive agent over another. In other severe myositis conditions, it is usual practice to start with high-dose corticosteroids for immediate action, followed by other non-steroidal immunomodulatory agents, such as methotrexate, azathioprine, rituximab and mycophenolate, under specialist guidance. Anti-HMGCR antibody level tends to correlate with disease activity, and decreasing level suggests resolving muscle damage. Long-term treatment may be warranted, with the tapering of the dose once the myonecrosis completely resolves; however, cases of resurgence of CK levels have been reported even without statin re-exposure.^62^

## Conclusions

Muscular side effects due to statins are likely to be over-reported, with the nocebo effect being responsible for a significant proportion of cases. Identifying and treating reversible risk factors associated with muscular symptoms can prevent the unnecessary discontinuation of statin therapy. A structured approach is recommended for the assessment of SRM so that statin discontinuation can be kept to a minimum. Every opportunity to restart statin therapy should be taken, and even a low-dose statin regimen may offer a significant reduction in risk for those with high baseline cardiovascular risk or pronounced hyperlipidaemia. The severity of the muscle damage should be correlated with CK elevation, but symptom correlation is poor. Severe cases of immune-mediated myonecrosis require specialist input. It should be stressed that such cases are very rare and should not deter physicians from advocating statin therapy for routine primary or secondary prevention. Longer-term management of dyslipidaemia remains a challenge in patients who have experienced statin-associated muscular side effects, but this can be mitigated by a better understanding of predisposing risk factors and through the advent of an increasing number of non-statin cholesterol-lowering therapies.
